# Digoxin-specific antibodies: a novel dosing strategy

**DOI:** 10.1007/s12471-023-01814-y

**Published:** 2023-10-20

**Authors:** Marieke A. Dijkman, Femke M. J. Gresnigt, Dylan W. de Lange

**Affiliations:** 1 Dutch Poisons Information Centre, University Medical Centre Utrecht, Utrecht, The Netherlands; 2 OLVG Hospital, Amsterdam, The Netherlands; 3 Department of Intensive Care Medicine, University Medical Centre Utrecht, Utrecht, The Netherlands

**Keywords:** Digoxin poisoning, Antidote, Digoxin-specific antibodies

## Abstract

Digoxin-specific antibodies (digoxin-Fabs) are of value in the treatment of a strongly suspected or a known, potentially life-threatening digoxin toxicity. These antibodies are not registered for use in Europe; therefore Dutch hospital pharmacies are not allowed to keep them in stock. In the Netherlands, digoxin-Fabs are stored in a national calamity stock of emergency medicines at the National Institute for Public Health and the Environment. In the case of a medical emergency, digoxin-Fabs are available after contact with the Dutch Poisons Information Centre. Recent studies have shown that the dose of digoxin-Fabs required to effectively treat digoxin toxicity is lower than previously thought. In this article, we present the adjusted digoxin-Fab dosing strategy currently recommended by the Dutch Poisons Information Centre (www.vergiftigingen.info). This new dose titration strategy is safe and effective and has a cost-saving side-effect.

## Background

Digoxin-specific antibodies (digoxin-Fabs) were first developed in 1967 for analytical purposes [1]. In 1976, Smith and colleagues successfully treated a patient who had ingested 22.5 mg digoxin with such digoxin-Fabs after conventional therapy had failed [2]. Ten years later, with only limited pharmacokinetic data and without randomised placebo-controlled trials having been performed, the first digoxin-Fab product was approved by the US Food and Drug Administration [3, 4]. Currently, digoxin-Fabs are indicated in the treatment of (potentially) life-threatening digoxin toxicity or digoxin overdose, with limited adverse effects (Tab. 1; [3, 5–7]).

**Table 1:** Indication criteria for treatment with digoxin-specific antibodies (digoxin-Fabs) [6, 7, 13]

*In acute poisoning based on ingested digoxin dose*
– Adults: > 10 mg in healthy adults, with a lower threshold in patients with renal insufficiency or underlying heart disease* – Children: > 4 mg (or > 0.1 mg/kg)
*In acute and chronic poisoning based on clinical features*
– Haemodynamic instability or life-threatening dysrhythmia, with elevated steady-state serum digoxin concentration > 2.6 nmol/l (2 ng/ml) a. Progressive (severe) sinus bradycardia (40–60/min) or second- or third-degree heart block unresponsive to atropine b. Increased automaticity (e.g. multiple ventricular ectopics or supraventricular tachyarrhythmias), severe ventricular tachycardia or ventricular fibrillation – Presence of manifestations of digoxin toxicity along with serum potassium > 5.0 mmol/l (5 mEq/l) a. Hyperkalaemia (K > 5.0 mmol/l) is a clinical marker for acute digoxin toxicity requiring treatment. This criterion may not be useful in chronic toxicity for several reasons, e.g. renal failure, or drugs interfering with the homeostatic mechanisms for excretion of potassium through the renin/angiotensin/aldosterone axis
*In acute and chronic poisoning based on serum digoxin concentrations*
– Independent of clinical features: a. Serum digoxin concentration ≥ 13 nmol/l (10 ng/ml) soon after ingestion in acute poisoning b. Serum digoxin concentration > 10 nmol/l (7.8 ng/ml) 6 h post-ingestion c. Serum digoxin concentration > 7.7 nmol/l (6 ng/ml) in chronic poisoning (adults) d. Serum digoxin concentration > 5.1 nmol/l (4 ng/ml) in chronic poisoning (children)

*A lower treatment threshold is recommended for patients older than 60 years of age, as they are at greater risk of mortality from digoxin poisoning, especially in the setting of other co-morbidities [6]

Back in 1986, the emphasis was on rapid, full neutralisation of all digoxin molecules. The total body burden of digoxin was estimated using the ingested digoxin dose or the plasma digoxin concentration and hence an equimolar dose of digoxin-Fab was recommended [2]. However, pharmacokinetic analysis of patients treated with digoxin-Fabs showed that, if digoxin-Fabs were infused during a short time period, about half of the infused digoxin-Fabs were cleared from the plasma before it could bind digoxin that was still dissociating from the tissues [8]. Therefore, it is recommended in current guidelines that half of the calculated equimolar loading dose be administered and, if a clinical response is not seen within 1–2 h, another half equimolar dose should be given [5]. An even more accurate dosing strategy is possible if the pharmacokinetics of digoxin and digoxin-Fabs and the poisoning scenario (acute versus chronic) are taken into account [7]. In particular, the rate at which digoxin is (re-)distributed in the presence of digoxin-Fabs is the key to the efficacy of digoxin-Fab dosing. Hence, administration of a small bolus, repeated if necessary, titrated against clinical effect has been suggested. Accumulating evidence is now available to justify this new approach, which might also have a cost-saving effect, as one vial of DIGIFab (BTG International Inc.) costs approximately 1500 € (2022 price) [7, 9].

## Mechanism of action

Digoxin toxicity is dose-dependent. A vial of 40 mg digoxin-Fab is capable of neutralising 0.5 mg digoxin [5]. Digoxin-Fabs predominantly bind digoxin in the circulation, but they may also diffuse into the interstitial space, binding digoxin there. The affinity of digoxin to digoxin-Fabs is 100- to 1000-fold higher than for its target, the Na^+^/K^+^-ATPase pump [7], causing digoxin to dissociate from its binding sites with a dissociation half-life of about 1 h [10]. A concentration gradient is established, which facilitates movement of dissociated digoxin into the interstitial and intravascular space [7, 8, 11]. Ventricular arrhythmias usually start to resolve within a few minutes after digoxin-Fab administration and, in general, the time required for reversal of digoxin-induced rhythm and conduction disturbances is reported to be 30–45 min [7, 11].

After digoxin-Fab administration, free and unbound serum digoxin concentrations will drop to near zero. However, the total serum digoxin concentration will increase rapidly (8- to 20-fold in patients with normal kidney function and up to 33-fold in those with impaired kidney function [12]), reflecting pharmacologically inactive digoxin bound to digoxin-Fabs and the continuous binding of redistributed digoxin from extravascular tissue stores to the plasma [2, 4, 7]. Free digoxin serum concentrations remain low for a considerable period of time, depending on the digoxin-Fab dose, infusion technique and the patient’s renal function [7]. A rebound in free digoxin serum concentration can be seen 12–24 h after digoxin-Fab administration in patients with normal renal function, and may be delayed up to 24–96 h in patients with renal dysfunction and on average 130 h in end-stage renal disease [4, 7]. Fortunately, this amount of free digoxin redistributed from the tissues may not necessarily reflect a high tissue concentration or cause cardiac toxicity [4].

Dissociation of the digoxin/digoxin-Fab complex in the intravascular system is not expected over extended periods of time [4, 7, 11], as the affinity constant (K_a_) between digoxin and digoxin-Fab is in the order of 10^10^ M^−1^ [11].

The elimination kinetics of the digoxin/digoxin-Fab complex is largely dependent on renal function, as glomerular filtration is permitted because the molecular weight of the digoxin-bound complex is below 60,000 Da [2, 10, 11]. In patients with renal failure, the plasma half-life of both digoxin (mean: 40 h) and digoxin-Fabs (15–20 h) is prolonged to over 100 h [5, 7]. In these patients, digoxin/digoxin-Fab complexes remain detectable in plasma for 2–3 weeks after digoxin-Fab administration [4, 11], but extracorporeal treatment for removal of the digoxin/digoxin-Fab complexes is not necessary [12]. In addition to the kidney, the reticuloendothelial system, which includes the liver, spleen and lymph nodes, is a potential site for catabolism of the digoxin/digoxin-Fab complexes [10, 11].

## Digoxin-Fab dosing in acute poisoning

Acute poisoning might occur in patients who are digoxin naïve or in patients that have been prescribed digoxin for a while. In patients who present early (< 6 h) after ingestion, the initial plasma digoxin concentration may be very high and overestimates the total body burden/load, as full tissue distribution has not yet occurred. Since digoxin has a slow onset of action, several hours may pass before the full toxicological impact of the digoxin overdose is seen. The critical question from a management point of view is how much digoxin needs to be neutralised to reduce toxicity. Since a therapeutic quantity of digoxin will have little effect in a digoxin-naive individual, complete neutralisation is unnecessary, and it might even be undesirable in patients on therapeutic digoxin, potentially worsening underlying medical conditions [7, 8].

**Fig. 1:**
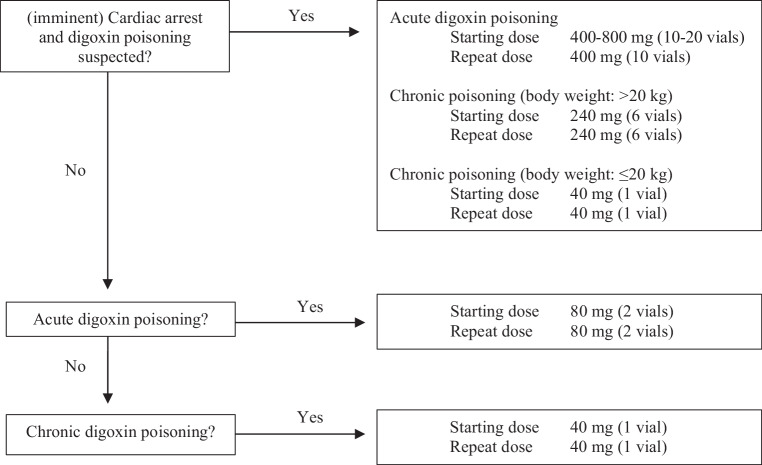
Dose titration regimens [5–7, 15] for digoxin-specific antibodies (digoxin-Fabs). In the case of an inadequate response, recurrence of symptoms, or clinical deterioration. One vial contains 40 mg of purified digoxin-Fab [5]

Pharmacokinetic simulations suggested that 40–80 mg of digoxin-Fab is sufficient to neutralise the amount of digoxin in the circulation in most acute overdoses [7]. After digoxin-Fabs have been administered, the total digoxin concentration in the circulation will increase and cannot be used to guide the dosing strategy further unless free digoxin can be measured. However, the onset of clinical improvement can be estimated based on electrocardiographic findings, haemodynamic status and potassium concentrations, and should be apparent within 60 min after administration of digoxin-Fabs. A further 80-mg dose is suggested after 60 min or earlier in the case of an inadequate response, recurrence of symptoms or clinical deterioration ([7]; Fig. 1). Titration as a dosing strategy for digoxin-Fabs results in a dose reduction of 65–75% compared to the old equimolar dosing strategy [9].

## Digoxin-Fab dosing in chronic poisoning

Chronic poisoning occurs as a result of either a patient repeatedly taking more than the prescribed dose, or of altered pharmacokinetics (e.g. renal impairment) in a patient taking the prescribed dose [8]. Most patients taking digoxin are older and have co-morbidities, like atrial fibrillation, congestive heart failure, diabetes and/or hypertension, and often receive drugs such as beta-adrenoceptor blockers, calcium antagonists and diuretics as co-medication [13]. The diagnosis of chronic digoxin toxicity can be particularly difficult because symptoms thought to be consistent with digoxin toxicity are potentially multifactorial and high serum digoxin concentrations are often a reflection of another problem, such as renal impairment [7, 13, 14]. Therefore, the indications for digoxin-Fabs are less well defined, but patients with ventricular tachyarrhythmia and cardiac arrest should be treated with digoxin-Fabs immediately [13]. In patients with chronic digoxin poisoning, full distribution has occurred and large quantities of digoxin will be outside the intravascular compartment [8]. Full neutralisation is not necessary and even undesired, as most patients have been prescribed digoxin for therapeutic purposes, and it may worsen underlying congestive heart failure or atrial fibrillation [3, 7]. It is advised that 40 mg digoxin-Fab be administered and that patients be observed for a clinical response. It should be noted that in chronic digoxin toxicity digoxin-Fabs may not be effective in reversing bradydysrhythmia or hyperkalaemia, for which conventional treatment is essential [13, 14]. If no clinical response is observed after 60 min, a second dose of 40 mg may be administered, to neutralise re-distributed digoxin [7]. In general, a total dose of 40–120 mg is considered sufficient [7, 14]. Chan et al. observed a comparable mortality rate and length of hospital stay in patients with chronic digoxin poisoning routinely treated with digoxin-Fabs and in those receiving supportive care [13]. The latter group only received digoxin-Fabs in the case of ventricular tachyarrhythmias. This suggests that in the majority of these patients death might be attributed to other illnesses or the patients’ general frail condition.

## Digoxin-Fab dosing in cardiac arrest

In the event of (imminent) cardiac arrest due to ventricular tachyarrhythmias and ventricular fibrillation, larger doses that will achieve full neutralisation are reasonable due to the uncertain aetiology of the medical emergency [6, 7]. In case the estimated digoxin dose or serum digoxin concentration is not available, empiric established doses of 400–800 mg for adults and children with acute poisoning and 240 mg for adults (patients > 20 kg) or 40 mg for children (< 20 kg) with chronic poisoning are recommended [5, 6, 15].

## Conclusion

Digoxin-specific antibodies are of value in the treatment of digoxin toxicity. A dose titration strategy avoids a mismatch between the timing of digoxin-Fab administration and the redistribution of digoxin from the tissues. For acute poisoning, digoxin-Fabs are effective. For chronic poisoning digoxin-Fabs are justified in the case of ventricular tachyarrhythmias but are otherwise considered less effective, as symptoms thought to be consistent with digoxin toxicity are potentially multifactorial and often less likely caused by digoxin alone. The adjusted digoxin-Fab dosing strategy, currently recommended by the Dutch Poisons Information Centre, has a cost-saving side-effect.
